# Monte Carlo-based scatter correction for the SMARTZOOM collimator

**DOI:** 10.1186/s40658-020-00318-7

**Published:** 2020-07-22

**Authors:** Martijn M. A. Dietze, Britt Kunnen, Martina Stella, Hugo W. A. M. de Jong

**Affiliations:** 1 Radiology and Nuclear Medicine, University Medical Center Utrecht, Utrecht University, P.O. Box 85500, 3508 GA Utrecht, The Netherlands; 2 Image Sciences Institute, University Medical Center Utrecht, Utrecht University, P.O. Box 85500, 3508 GA Utrecht, The Netherlands

**Keywords:** Monte Carlo, SPECT, Reconstruction, Scatter, Correction, Myocardial, Perfusion

## Abstract

**Background:**

Myocardial perfusion imaging is a commonly performed SPECT protocol and hence it would be beneficial if its scan duration could be shortened. For traditional gamma cameras, two developments have separately shown to allow for a shortened scan duration: (i) reconstructing with Monte Carlo-based scatter correction instead of dual-energy window scatter correction and (ii) acquiring projections with the SMARTZOOM collimator instead of a parallel-hole collimator. This study investigates which reduction in scan duration can be achieved when both methods are combined in a single system.

**Results:**

The SMARTZOOM collimator was implemented in a Monte Carlo-based reconstruction package and the implementation was validated through image quality phantom experiments. The potential for scan duration reduction was evaluated with a phantom configuration that is realistic for myocardial perfusion imaging. The original reconstruction quality was achieved in 76 ± 8% of the original scan duration when switching from dual-energy window scatter correction to Monte Carlo-based scatter correction. The original reconstruction quality was achieved in 56 ± 13% of the original scan duration when switching from the parallel-hole to the SMARTZOOM collimator. After combining both methods in a single system, the original reconstruction quality was achieved in 34 ± 7% of the original scan duration.

**Conclusions:**

Monte Carlo-based scatter correction combined with the SMARTZOOM collimator can further decrease the scan duration in myocardial perfusion imaging.

## Background

Myocardial perfusion imaging is a commonly performed technetium-99 m (^99m^Tc) single-photon emission computed tomography (SPECT) protocol [1]. Scan duration reduction for this protocol has been a major research theme because potential time gain would allow for substantial increases in the scanner throughput. The duration of a scan can, e.g., be shortened through hardware design choices (e.g., by trying to increase the system sensitivity) or improvements in the reconstructed image quality (so that the original quality can be achieved with fewer counts) [2]. We will discuss two successful examples that have been introduced for traditional (i.e., general-purpose) gamma cameras.

First, the SMARTZOOM collimator has been introduced in clinical practice by Siemens Healthineers (Erlangen, Germany) on IQ-SPECT systems [3, 4]. This collimator has a converging (i.e., high sensitivity) inner part with diverging (low sensitivity) outer regions. By aiming the converging part of the collimator on the heart (in the “sweet spot”), up to 4 × more photons can be collected in comparison with a conventional parallel-hole collimator. The diverging parts of the collimator ensure that photons originating from outside of the heart will not produce truncation artifacts in the reconstruction. Since this collimator design provides a higher local sensitivity, it is frequently used to decrease the scan duration [5].

Second, the reconstruction image quality can be improved by implementing sophisticated models for the photon physics (e.g., point spread function modeling and attenuation correction) in the reconstruction algorithms. For myocardial perfusion imaging with a parallel-hole collimator, it has been shown that Monte Carlo-based scatter correction generates higher quality images (i.e., a higher contrast) when compared with the conventional dual-energy window scatter correction approach [6, 7] because the non-uniform nature of the attenuation is better incorporated and the reconstructions are not deteriorated by additional Poisson noise in the scatter estimate. The improved contrast can be combined with an increased smoothing (e.g., with a Gaussian filter) so that more noise can be accepted in the initial image, which subsequently also allows for a reduction in scan time.

We believe that it is possible to combine Monte Carlo-based scatter correction together with the SMARTZOOM collimator. This combination might lead to an even larger reduction in scan duration. This study will demonstrate the implementation of Monte Carlo-based scatter correction for the SMARTZOOM collimator and will determine the achievable scan duration reduction using phantom experiments.

## Methods

### Acquisition

All phantom scans were performed on a dual-head Siemens Symbia T2 SPECT/CT scanner with our current clinical scan protocol for myocardial perfusion imaging. Projections were acquired at a 128 × 128 grid with a 4.79-mm isotropic pixel size. The photopeak window captured photons from 129 to 150 keV, while the scatter window ranged from 108 to 129 keV. The attenuation map was created from the low-dose CT scan. The scanner acquired 34 projections (17 per detector head) over 208° (with a start angle of 54°) in step-and-shoot mode. The experiments were performed with two collimators:

#### Parallel-hole

The low-energy high-resolution (LEHR) parallel-hole collimator has a uniform sensitivity over its entire surface and is conventionally used for ^99m^Tc imaging. The collimator had a hole length of 24.05 mm, a hole diameter of 1.11 mm, and a septal thickness of 0.16 mm [8]. The detector heads performed a body-tracing orbit.

#### SMARTZOOM

**Fig. 1:**
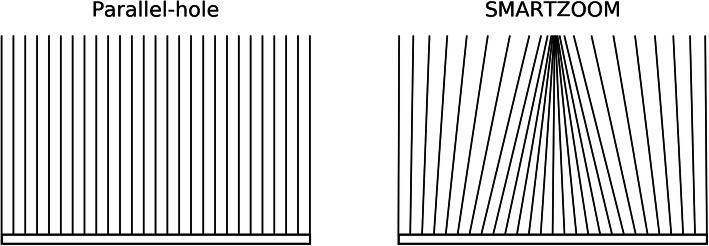
An illustration of the field of view of a conventional parallel-hole collimator and the SMARTZOOM collimator. The highest sensitivity is achieved in the regions with the highest line density

The SMARTZOOM collimator differs from the parallel-hole collimator by having a converging part in the middle of the collimator, while the outer parts are diverging (see Fig. 1 for an illustration). The converging part increases the sensitivity, while the diverging parts ensure that no truncation artifacts arise in the reconstruction. The hole length of the SMARTZOOM collimator was 40.25 mm, the inner hole diameter was 1.95 mm, and the septal thickness was between 0.2 and 0.4 mm [8]. The SMARTZOOM collimator is difficult to manufacture because of the complicated geometry and hence every SMARTZOOM collimator will have slightly different angles. These angles have been measured and this information is provided by the manufacturer. For the SMARTZOOM collimators that were present on the two detector heads of the SPECT/CT, the focal lengths were 54.5 and 53.2 cm. The detector heads performed a cardio-centric orbit with a fixed center-to-detector distance of 28 cm.

### Reconstruction

The phantom acquisitions were reconstructed with our in-house developed maximum-likelihood expectation-maximization (MLEM) reconstruction software package Utrecht Monte Carlo System (UMCS), which has been validated for a variety of isotopes [9, 10]. UMCS accounts for photon attenuation via correction with the attenuation map (obtained from the low-dose CT) and for collimator resolution via distance-dependent point spread function (PSF) modeling [11]. The performance of two scatter correction methods was compared.

#### Dual-energy window

Dual-energy window scatter correction, in which the scatter window projections are added in the reconstruction loop at a certain scatter multiplier factor [12], is conventionally used in clinical reconstruction software because of its straightforward implementation. This approach, however, has two drawbacks. First, the scatter multiplier factor is chosen to represent an average scatter medium (often, and also used in this study: *k* = 0.5), which leads to sub-optimal performance for patients with a non-average physique (e.g., with a very small or large body-mass index). And second, the scatter projections are corrupted by Poisson noise, which increases the noise level in the reconstruction. This latter effect can (partly) be compensated for by filtering the projections, but the optimal filtering magnitude to apply depends heavily on the phantom configuration and was hence not employed in this study.

#### Monte Carlo-based

A more sophisticated method for scatter correction is to simulate the physical interactions of the photons in the patient. This is performed by Monte Carlo-based sampling of photon emissions from the intermediate reconstructed activity image and determining their traveling distance, traveling angle, and energy loss when an interaction (at a specific scatter position) with the scattering medium (the attenuation map) occurs. This process is hence repeated for 2^nd^, 3^rd^, etc. orders of scatter until the photon is either absorbed or falls below an energy limit. The photon physics simulation can take a long time but is greatly accelerated with variance reduction techniques. Convolution-based forced detection is employed for this purpose in UMCS. In convolution-based forced detection, at every scatter position, the probability is calculated that the photon scatters towards the detector and is detected. In addition, instead of adding one detection event to the projection, all the potential events are projected using a predefined and tabulated kernel. It was previously determined that the change in the simulated projections became very small after the simulation of 5 × 10^6^ photons [13] and hence this number of simulated photons was used per projection. The simulated scatter projections are added in the reconstruction loop similarly as is done in the dual-energy window approach.

The variable field of view of the SMARTZOOM collimator was implemented in UMCS by warping the intermediate reconstruction volumes and the attenuation map during the forward and backward projection step of the reconstruction [14] (using the collimator hole angles that are provided by the scanner manufacturer) so that the rays under an angle become parallel to the detector. With this approach, it becomes possible to perform the further forward and backward projection operations (e.g., weighted summing of the image volume to obtain the projection) the same as is done with parallel-hole collimators. This approach is relatively easy to implement and has the additional benefit that only a single point spread function (as a function of the source-to-detector distance) is required since the detector response is approximately shift-invariant for translations in the detector plane (after warping) [15]. Since the septal thickness of the SMARTZOOM collimator ranged between 0.2 and 0.4 mm, point spread functions were created for an average septal thickness of 0.3 mm.

### Validation

To verify whether our reconstruction implementation of the SMARTZOOM collimator was accurate, we compared the reconstructions obtained from UMCS (with dual-energy window correction) with reconstructions from the clinical software. Two image quality phantoms were used for this task:

- A rack with five point sources (positioned in the same plane) of approximately 29 MBq ^99m^Tc each that was scanned with 30 s per projection. This phantom was used to check whether the point sources were reconstructed to the same position (validating the warping methodology) and to determine whether they were reconstructed as small point sources (validating the point spread function implementation).
- A cylinder (6820 mL volume) filled uniformly with 148 MBq ^99m^Tc that was scanned with 30 s per projection. This phantom was used to evaluate the uniformity of the reconstruction (validating the attenuation correction approach) and the reconstruction noise patterns (which are dependent on the used reconstruction algorithm).

The clinical reconstructions employed the MLEM reconstruction algorithm for the parallel-hole collimator but used the ordered subset conjugate gradient minimizer (OSCGM) for the SMARTZOOM collimator. The reconstructions were, for both collimators, made with 30 iterations. No subsets were used because of the relatively low number of projection angles available.

The UMCS reconstructions used the MLEM reconstruction algorithm for both collimators. The respective iteration numbers were chosen by visually determining the best-matching images with the clinical reconstructions. This resulted in 40 iterations for the parallel-hole collimator and 250 iterations for the SMARTZOOM collimator. The UMCS reconstructions for the SMARTZOOM collimator required substantially more iterations than the clinical software due to faster convergence of the OSCGM algorithm compared to MLEM [16].

Both the clinical and the UMCS reconstructions were made with attenuation correction, point spread function modeling, and dual-energy window scatter correction. The reconstructions were made on a 128 × 128 × 128 grid with a 4.79-mm isotropic pixel size and a 5-mm full-width at half-maximum (FWHM) Gaussian post-reconstruction filter was applied.

### Scan duration reduction

The potential for scan duration reduction was evaluated for the primary task in myocardial perfusion imaging: the detection of a cardiac defect. The most accurate detection can be made when (i) the contrast between the healthy tissue and the defect is as large as possible and (ii) the noise in the background is as low as possible. The reconstructions of the collimator and scatter correction combinations (made with 100 iterations and no post-reconstruction filter) will hence be compared on a metric that combines both of these measures: the contrast-to-noise ratio (CNR).

#### Contrast

**Fig. 2:**
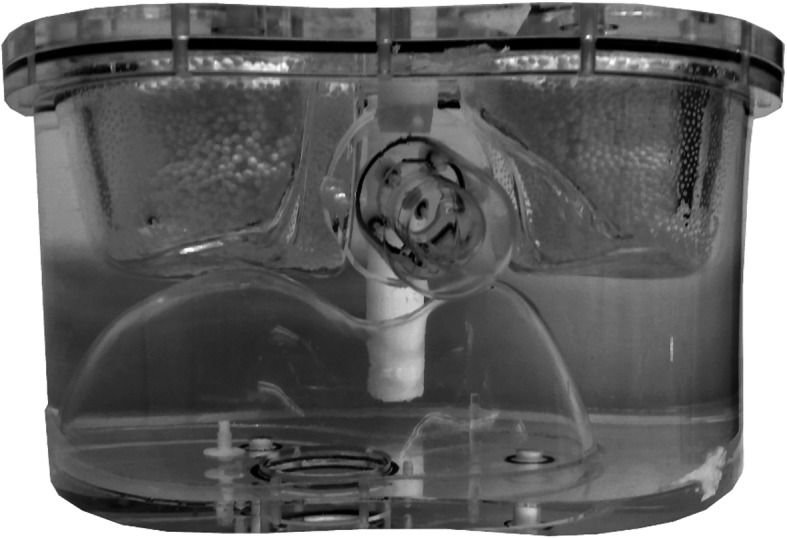
The anthropomorphic phantom that comprises a cardiac insert with a defect

The contrast was determined in an anthropomorphic phantom (model ECT/TOR/P) that comprised a cardiac insert (model ECT/CAR/I) with a defect of 2 mL (i.e., with no activity present) (see Fig. 2). The phantom was filled with a ^99m^Tc activity distribution that is clinically encountered in myocardial perfusion imaging (see Table 1) [5, 17]. The phantom was scanned with a relatively large acquisition time of 90 s per projection to obtain low-noise projections. The hot wall and the defect were manually delineated on the SPECT reconstructions and the contrast was calculated as follows:

$$ \mathrm{C}=\frac{I_{\mathrm{w}}-{I}_{\mathrm{d}}}{I_{\mathrm{d}}} $$

**Table 1:** The organ activity concentrations in the myocardial perfusion phantom

	Volume [mL]	Concentration [kBq/mL]	Activity [MBq]
Liver	1160	94	109.0
Background	8838	7.6	67.2
Heart	118	159	18.8

where *I*_w_ is the intensity in the hot wall and *I*_d_ is the intensity in the defect.

#### Noise

The noise (calculated as the standard deviation in the volume of interest) should ideally be determined in a relatively large volume that is not corrupted by resolution effects. Normally, one could, e.g., determine the noise in the background volume of the anthropomorphic phantom. For the SMARTZOOM collimator, however, we are only interested in the noise that is present in the “sweet spot” and hence this phantom could not be used.

Instead, the noise was determined in the “sweet spot” of the cylinder phantom (that was used for the reconstruction validation) with its total number of counts scaled to the background activity concentration of the myocardial perfusion phantom at a clinical activity level (100 MBq, from 800 MBq injected activity in combination with 8 gating frames) when scanned with a clinical scan duration (20 s per projection). These sampled projections were created by putting all events of the original projections in a list, performing a random shuffle of the list, and taking the first 12% of the events. Since the SPECT reconstructions are sensitive to noise patterns for these low-count projections, the sampling and reconstruction process was repeated 20 times to obtain statistically significant results.

#### Contrast-to-noise

Normally, the CNR is determined at a set number of iterations. In this work, however, this is not feasible since the convergence speed between the several collimator and scatter correction combinations varies substantially. Instead, the maximum achieved CNR over the iterations was collected so that the convergence speed was no longer of influence.

To determine the scan duration reduction that can be achieved, the scaled cylinder projections were further sub-sampled to obtain projections that would be obtained with 95 to 5% (in steps of 5%) of the original scan duration. The maximum CNR values were collected for every scan duration and it was determined at which scan durations an equal reconstruction quality was achieved.

## Results

### Validation

**Fig. 3:**
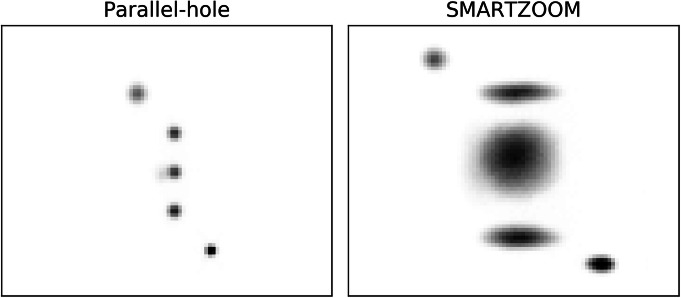
Projections of the five ^99m^Tc point sources obtained with the parallel-hole and the SMARTZOOM collimator

Projections of the ^99m^Tc point sources obtained with the parallel-hole and the SMARTZOOM collimator are shown in Fig. 3. The point spread function of the SMARTZOOM collimator has a spatial variance over the collimator surface: the PSF is largest in the middle of the collimator (where both directions converge) and smallest in the collimator corners (where both directions diverge). The remaining areas have an asymmetric PSF since these parts have convergence in one direction and divergence in the other.

**Fig. 4:**
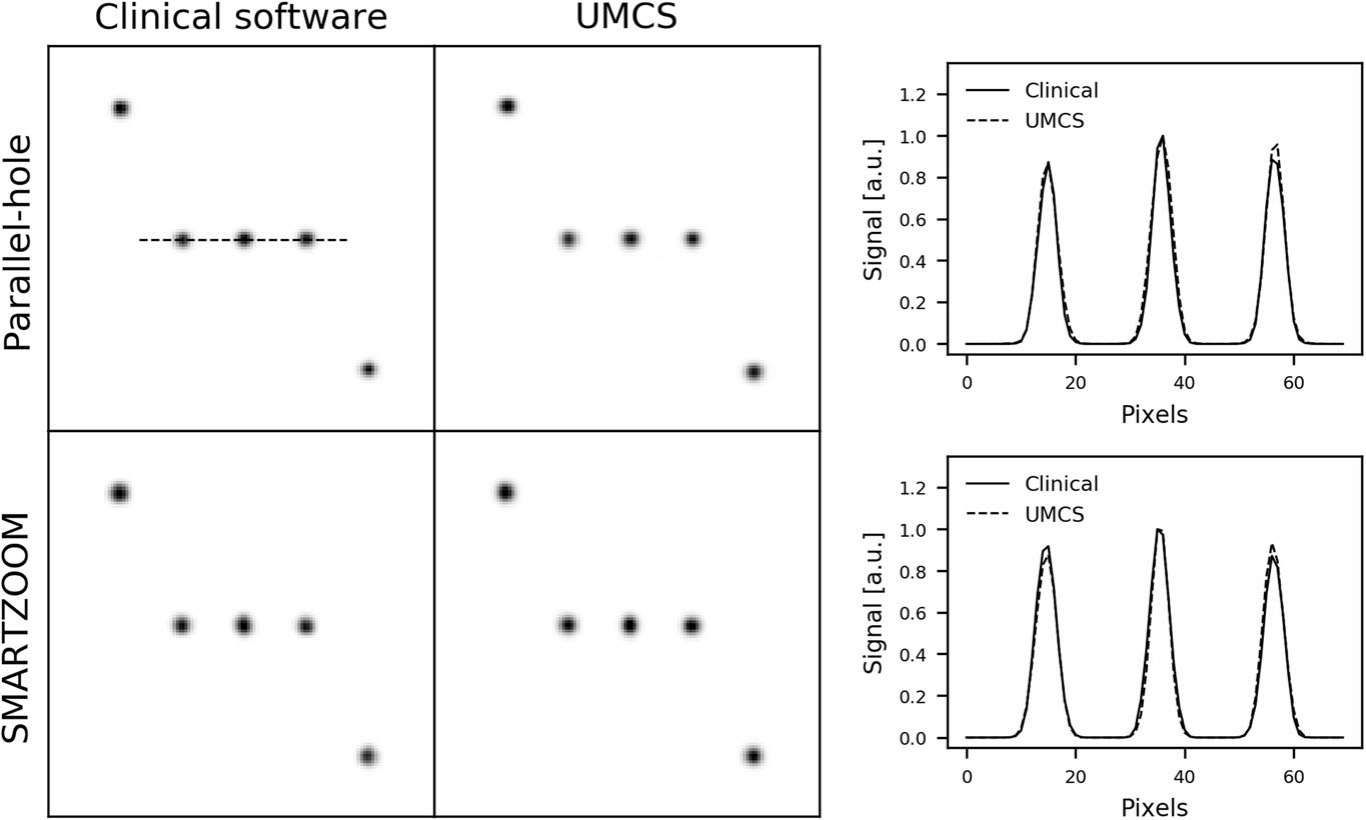
The reconstructions of the point sources obtained with the parallel-hole and the SMARTZOOM collimator when reconstructed with the clinical software and UMCS shown together with the profile at the location of the dashed line in the upper left reconstruction

The reconstructions of the point sources are shown in Fig. 4 together with their profiles. The point sources reconstructed to the same location and were approximately the same size. This result demonstrates that the warping and the point spread function modeling were correctly implemented for the SMARTZOOM collimator geometry.

**Fig. 5:**
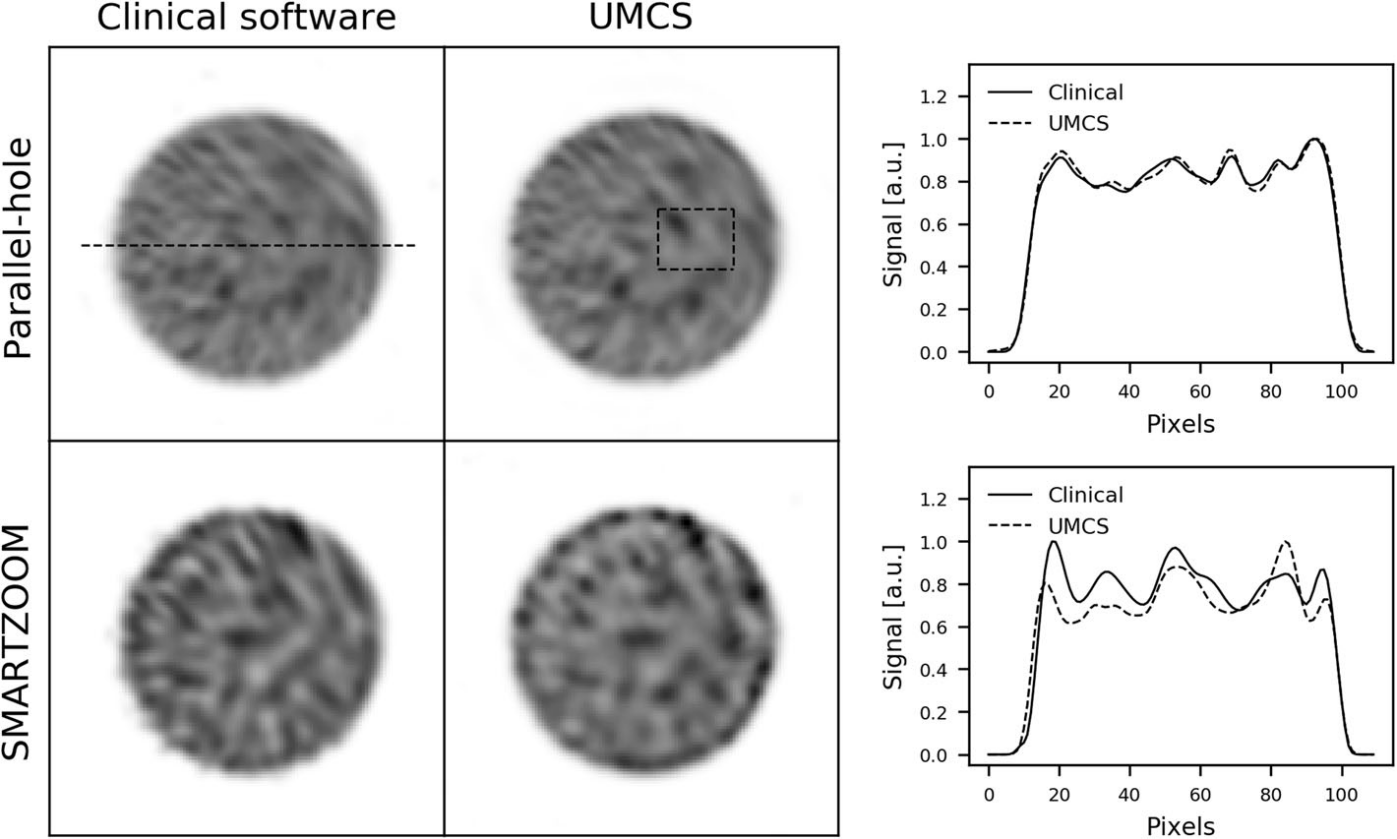
The reconstructions of the uniformly filled cylinder obtained with the parallel-hole and the SMARTZOOM collimator when reconstructed with the clinical software and UMCS shown together with the profile at the location of the dashed line in the upper left reconstruction. The dashed rectangle in the upper right reconstruction represents the volume in which the noise was calculated for the CNR analysis

The reconstructions of the uniformly filled cylinder are shown in Fig. 5 together with their profiles. The noise patterns are similar in the clinical software and UMCS in the case of the parallel-hole collimator. For the SMARTZOOM collimator, the patterns were different because of the different reconstruction algorithms used (OSCGM versus MLEM). All reconstructions showed a uniform distribution, which demonstrates that the attenuation correction was correctly implemented. We conclude that our implementation of the SMARTZOOM collimator is accurate.

### Scan duration reduction

#### Contrast

**Fig. 6:**
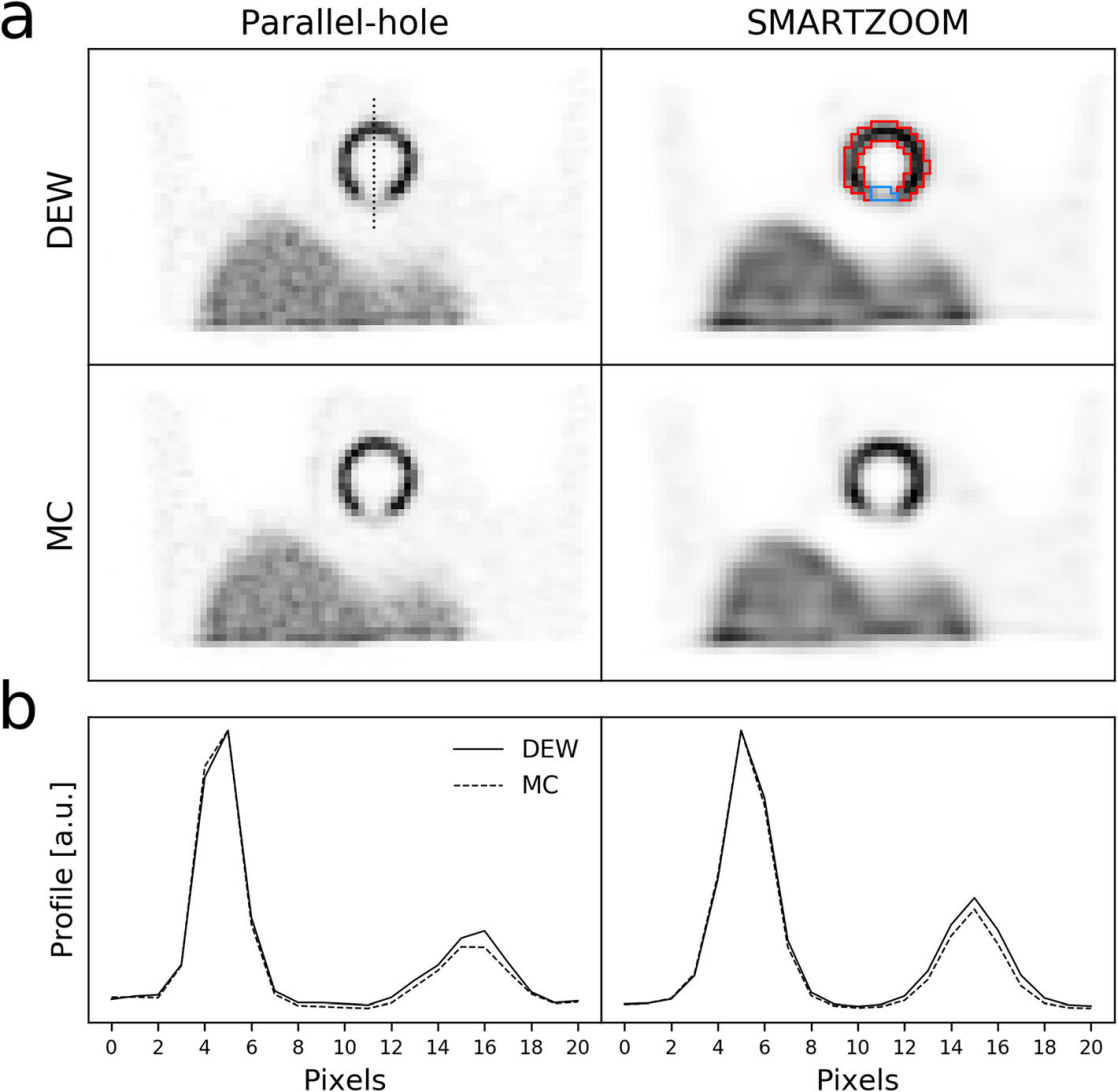
**a** The reconstructions of the myocardial perfusion phantom at iteration 100 obtained with the parallel-hole and the SMARTZOOM collimator when reconstructed with dual-energy window and Monte Carlo-based scatter correction. The dotted line in the upper left reconstruction represents the location of the profiles. The red lines in the upper right reconstruction represent the mask of the hot wall and the blue lines represent the mask of the defect. **b** The profiles over the cardiac insert. The left and the right (lower) peaks are the hot wall and the defect, respectively

The reconstructions of the myocardial perfusion phantom at iteration 100 are shown in Fig. 6 for all collimator and scatter correction combinations, together with the profiles over the cardiac insert with the defect. The profiles illustrate that Monte Carlo-based scatter correction achieves a slightly better contrast (difference in intensity between the hot wall and the defect) than dual-energy window scatter correction for both the parallel-hole and the SMARTZOOM collimator.

**Fig. 7:**
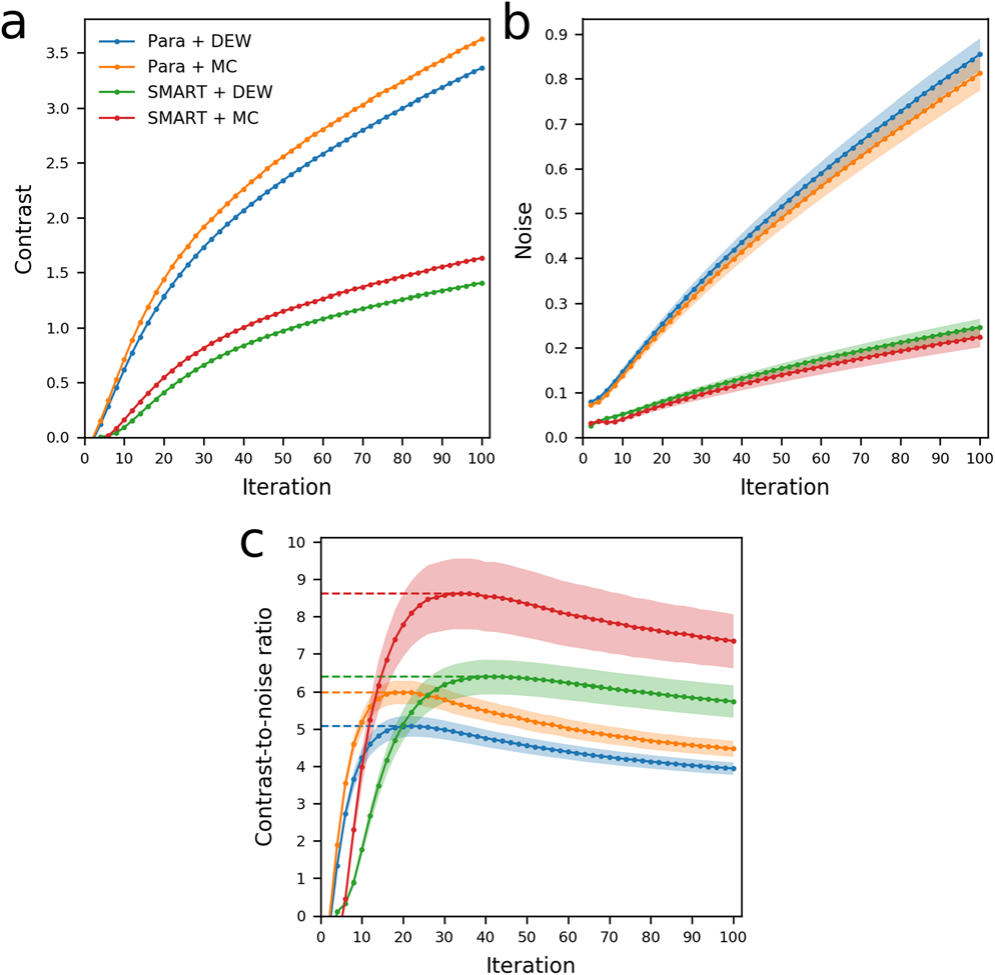
The **a** contrast, **b** noise, and **c** contrast-to-noise ratios, as a function of the iteration number. The shaded regions in **b** and **c** represent the standard deviation over the 20 noise realizations. The dotted lines in **c** indicate the maximum achieved CNR values

The contrast, as a function of the iteration number, is shown in Fig. 7a. This figure illustrates that the SMARTZOOM collimator has a lower contrast than the parallel-hole collimator at the same iteration. This occurs because of the slower convergence of the SMARTZOOM collimator (due to the intermediate warping during the reconstruction) and because of the worse resolution (due to the relatively larger detector-source distance).

#### Noise

The noise, as a function of iteration number, is shown in Fig. 7b. The SMARTZOOM collimator has a lower noise level than the parallel-hole collimator because it captures up to 4 × more photons in the “sweet spot” and because it convergences slower. The reconstructions made with Monte Carlo-based scatter correction result in a slightly lower noise level than those made with dual-energy window scatter correction. This is explained by the almost noiseless scatter projections in the case of Monte Carlo-based scatter correction, whereas the scatter projections are corrupted by Poisson noise in the case of dual-energy window correction.

#### Contrast-to-noise

The contrast-to-noise ratio, as a function of iteration number, is shown in Fig. 7c. The CNR reaches a maximum value for all collimator and scatter correction combinations: after this point, the increase in reconstruction noise starts to dominate over the further gain in contrast. The maximum value is achieved at a different iteration number for every combination.

The best image quality, i.e., the highest CNR, is achieved for the SMARTZOOM collimator with Monte Carlo-based scatter correction. The lowest image quality is achieved for the parallel-hole collimator with dual-energy window scatter correction.

**Fig. 8:**
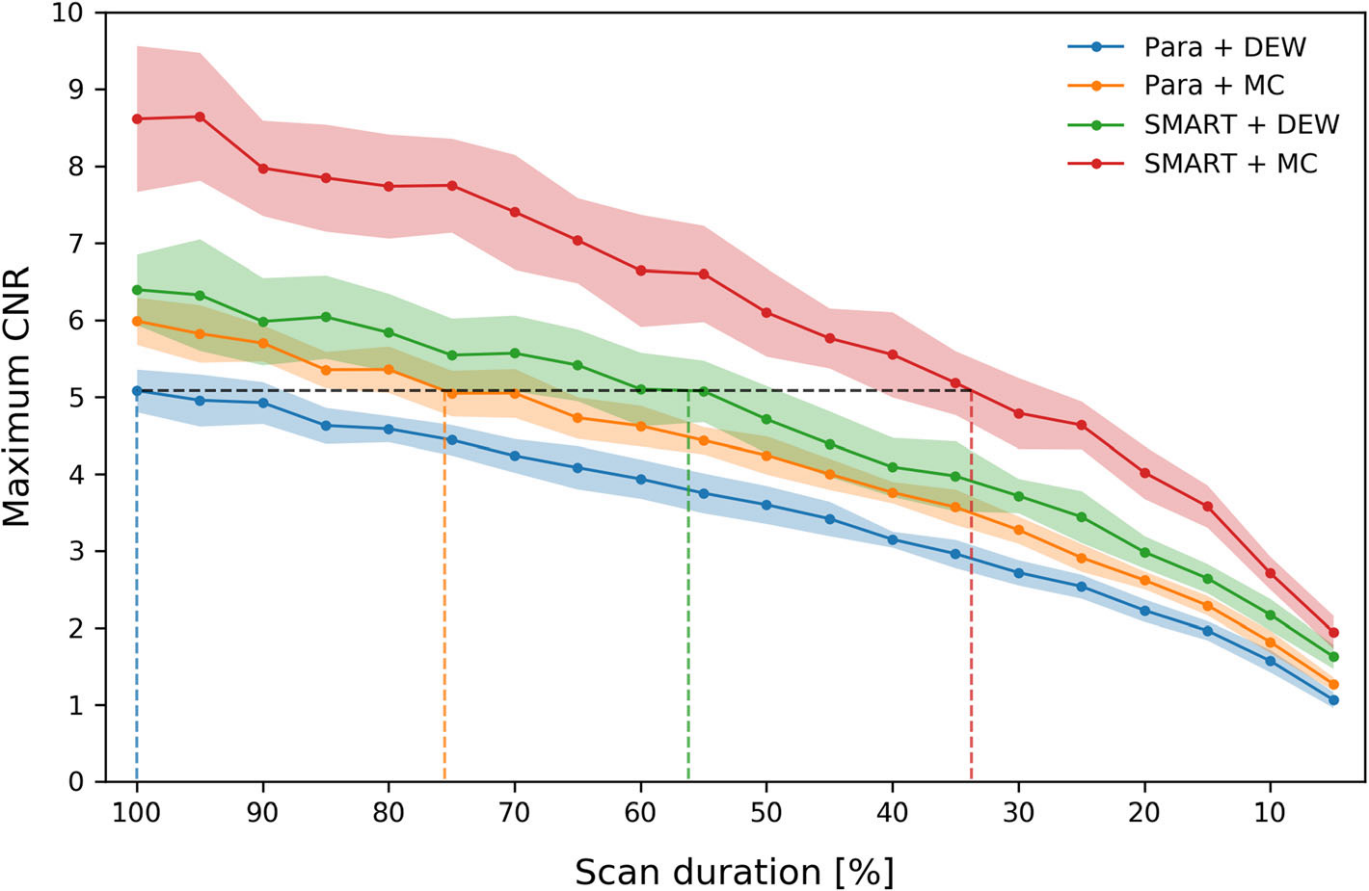
The maximum CNR, as a function of the scan duration. The black dashed line is drawn at the maximum CNR achieved by the parallel-hole collimator with dual-energy window scatter correction. The dashed lines in color are drawn at the scan durations at which the same maximum CNR is achieved for the other collimator and scatter correction combinations. The shaded areas represent the standard deviation over the 20 noise realizations

The maximum achieved CNR, as a function of the scan duration, is shown in Fig. 8. The maximum CNR decreases when the scan duration is shortened since the projection noise becomes greater. It was determined at which scan duration the same reconstruction quality as that of the parallel-hole collimator with dual-energy window scatter correction was achieved. This resulted in the following results:

- By moving from dual-energy window to Monte Carlo-based scatter correction, the same reconstruction quality was achieved in 76 ± 8% of the original scan duration.
- By moving from the parallel-hole to the SMARTZOOM collimator, the same reconstruction quality was achieved in 56 ± 13% of the original scan duration.
- By combining Monte Carlo-based scatter reconstruction and the SMARTZOOM collimator in a single system, the same reconstruction quality was achieved in 34 ± 7% of the original scan duration.

## Discussion

This study investigated the potential for Monte Carlo-based scatter correction in combination with the SMARTZOOM collimator for myocardial perfusion imaging and demonstrated that the scan duration could be shortened even further.

There are three limitations to the current study. First, only one cardiac phantom configuration was studied. The relative performance of the collimator and scatter correction combinations will be different when other activity distributions are imaged. The parallel-hole collimator will perform relatively better when smaller defects need to be visualized (due to its better resolution). The SMARTZOOM collimator will perform relatively better when the activity injected in the patient is lower (due to its better sensitivity). Second, only the contrast-to-noise ratio in the reconstructions was evaluated as a measure of detectability. In certain situations, it may be beneficial to have a better spatial resolution in the reconstruction rather than a lower noise level. And third, no clinical evaluation was performed. The performance in a clinical setting may be different than with phantom experiments because of patient movements (including respiratory and cardiac motion) and non-uniform activity distributions. For a full comparison between the parallel-hole and the SMARTZOOM collimator, a study needs to be performed that investigates more (clinical) activity distributions and calculates more measures of detectability.

There are various practical considerations when employing dual-energy window correction. The scatter multiplier factor can be set higher or lower, the scatter window can be set broader or narrower, and the scatter projection can be filtered or not. In this manuscript, the current clinical settings in our institute were used (scatter multiplier of *k* = 0.5, a scatter window from 108 to 129 keV, and no filtering of the scatter projections). For the scatter multiplier factor, it was calculated with the Monte Carlo-based scatter simulator that the optimal *k* values (based on the total number of counts) were 0.58 for the parallel-hole and 0.56 for the SMARTZOOM collimator. For the scatter window range and the filtering of the projections, the changes in these values will generally be a trade-off between reconstruction noise and contrast. The optimal settings for the dual-energy window settings will depend on the activity distribution configuration and cannot be optimized for every patient separately in clinical practice. An advantage of Monte Carlo-based scatter correction is that the above values do not need to be set.

The time required for reconstruction differs for the studied scatter correction and collimator combinations. A disadvantage of Monte Carlo-based scatter correction over window-based correction is that more computational power is required for the simulation of the photon physics. A disadvantage of the SMARTZOOM collimator over the parallel-hole collimator is that more computational power is required for the forward and backward warping steps during the reconstruction. In the current non-optimized implementation (using a single core on a 3.1 GHz processor), the time required for 100 iterations (and no subsets) was 70 min for the parallel-hole collimator with dual-energy window scatter correction, 108 min for the parallel-hole collimator with Monte Carlo-based scatter correction, 126 min for the SMARTZOOM collimator with dual-energy window scatter correction, and 153 min for the SMARTZOOM collimator with Monte Carlo-based scatter correction.

The difference in the myocardial perfusion phantom profiles between dual-energy window scatter correction and Monte Carlo-based scatter correction was relatively small. To evaluate whether the difference was significant, 20 projections were randomly sampled from the long projection (each projection containing 50% of the counts) and individually reconstructed. The contrast was calculated for every reconstruction and the average contrast with the associated standard deviation was determined. This investigation demonstrated that, in line with previous studies [6, 7], the contrast of Monte Carlo-based scatter correction was significantly higher (two-sided *t* test, *p* < 0.05) than that of dual-energy window correction.

The reconstructions made with Monte Carlo-based scatter correction had an improved contrast over the reconstructions made with dual-energy window scatter correction. We used this property to shorten the scan duration. However, there could also be value in having an increased reconstruction image quality, e.g., by being able to better detect smaller myocardial defects. An observer study would need to be performed to evaluate whether such sharper reconstructions indeed provide more diagnostic information.

Monte Carlo-based scatter correction was studied for the SMARTZOOM collimator because this was the only focusing collimator dedicated to cardiac imaging in our institute. However, we expect that other focusing collimators (such as the cone beam and the fan beam collimators) that are employed in cardiac imaging could similarly benefit from Monte Carlo-based scatter correction.

The implementation of realistic photon interaction physics in the forward projector does not only allow for improved scatter correction but also opens up the possibility of studies with more complicated photon spectra, such as in multi-isotope studies [18]. For instance, Du et al. [19] studied the performance of simultaneous ^99m^Tc/^123^I imaging in combination with the SMARTZOOM collimator. Such studies can be used to potentially gain more information on cardiac functioning.

## Conclusions

Monte Carlo-based scatter correction and the SMARTZOOM collimator have individually shown to accomplish a scan duration reduction in myocardial perfusion imaging. We demonstrated that their combination can provide an even further scan duration reduction.

## Data Availability

All data is stored at the University Medical Center Utrecht, Utrecht, The Netherlands.

## Abbreviations

CNR: Contrast-to-noise ratio
DEW: Dual-energy window
FWHM: Full-width at half-maximum
LEHR: Low-energy high-resolution
MC: Monte Carlo
MLEM: Maximum-likelihood expectation-maximization
OSCGM: Ordered subset conjugate gradient minimizer
PSF: Point spread function
SPECT: Single-photon emission computed tomography
UMCS: Utrecht Monte Carlo System
